# RAGE Controls Activation and Anti-Inflammatory Signalling of Protein C

**DOI:** 10.1371/journal.pone.0089422

**Published:** 2014-02-24

**Authors:** Natascha Braach, David Frommhold, Kirsten Buschmann, Johanna Pflaum, Lutz Koch, Hannes Hudalla, Kathrin Staudacher, Hongjie Wang, Berend Isermann, Peter Nawroth, Johannes Poeschl

**Affiliations:** 1 Department of Neonatology, University Children’s Hospital, Heidelberg, Germany; 2 Department of Clinical Chemistry and Biochemistry, Otto-von-Guericke-University, Magdeburg, Germany; 3 Department of Medicine I and Clinical Chemistry, University Hospital, Heidelberg, Germany; King’s College London School of Medicine, United Kingdom

## Abstract

**Aims:**

The receptor for advanced glycation endproducts, RAGE, is a multiligand receptor and NF-κB activator leading to perpetuation of inflammation. We investigated whether and how RAGE is involved in mediation of anti-inflammatory properties of protein C.

**Methods and Results:**

We analyzed the effect of protein C on leukocyte adhesion and transmigration in WT- and RAGE-deficient mice using intravital microscopy of cremaster muscle venules during trauma- and TNFα-induced inflammation. Both, protein C (PC, Ceprotin, 100 U/kg) and activated protein C (aPC, 24 µg/kg/h) treatment significantly inhibited leukocyte adhesion in WT mice in these inflammation models. The impaired leukocyte adhesion after trauma-induced inflammation in RAGE knockout mice could not be further reduced by PC and aPC. After TNFα-stimulation, however, aPC but not PC treatment effectively blocked leukocyte adhesion in these mice. Consequently, we asked whether RAGE is involved in PC activation. Since RAGE-deficient mice and endothelial cells showed insufficient PC activation, and since thrombomodulin (TM) and endothelial protein C receptor (EPCR) are reduced on the mRNA and protein level in RAGE deficient endothelial cells, an involvement of RAGE in TM-EPCR-dependent PC activation is likely. Moreover, TNFα-induced activation of MAPK and upregulation of ICAM-1 and VCAM-1 are reduced both in response to aPC treatment and in the absence of *RAGE*. Thus, there seems to be interplay of the RAGE and the PC pathway in inflammation.

**Conclusion:**

RAGE controls anti-inflammatory properties and activation of PC, which might involve EPCR and TM.

## Introduction

Protein C (PC) is synthesized by the liver, endothelial cells, leukocytes, and keratinocytes [1]. Binding of thrombin to thrombomodulin (TM) leads to activation of PC, amplified by the endothelial protein C receptor (EPCR) [2]. The aPC-TM-EPCR-complex activates protease-activated-receptor 1 (PAR-1) so that activated protein C (aPC) elicits potent anti-inflammatory and cytoprotective effects independent of aPC’s anti-coagulatory properties [1], [2]. In endothelial cells, activation of PAR-1 inhibits NF-κB translocation which results in a reduced production of pro-inflammatory cytokines and expression of cell adhesion molecules such as intercellular adhesion molecule 1 (ICAM-1) and vascular cell adhesion molecule 1 (VCAM-1) [3], and thereby blocks leukocyte recruitment, while signalling mechanism may differ in other cell types [4], [5]. The cascade of leukocyte recruitment plays a crucial role in the immune defense during inflammation [6]. Capture of free flowing leukocytes is followed by leukocyte rolling along the endothelial layer, triggering the activation of the β_2_-integrins which interact with different endothelial ligands such as ICAM-1 [7], [8]. This leads to firm adhesion to the inflamed endothelium and finally to leukocyte transmigration [6], [9].

Based on recent *in vivo* studies leukocyte recruitment can be blocked by aPC in various models of inflammation [10]–[12]. There is increasing evidence that this holds true for the zymogen protein C [13]–[15]. Despite the withdrawal of aPC for treatment of septic patients, the investigation of anti-inflammatory properties of PC and its underlying mechanisms is still of high interest to target pro-inflammatory pathways [16]–[19].

Many pro-inflammatory pathways are mediated by the transcription factor NF-κB which can also be activated by the pattern recognition receptor RAGE, receptor for advanced glycation end products [20]–[23]. As a multiligand receptor, RAGE binds to HMBG1, S100, CD 11b/CD18 (Mac-1) and others [7], [8], [24]–[27], serves as a signalling molecule in the innate immune system and is thereby involved in a variety of inflammatory diseases [28]–[32].

The fact that the PC pathway is involved in these conditions too, raised the question whether RAGE may contribute to the anti-inflammatory properties of PC through a yet to be defined mechanism. Therefore, we studied the effect of PC and aPC on leukocyte adhesion in *RAGE^−/−^* mice using intravital microscopy of cremaster muscle venules during trauma- and TNFα-induced inflammation, which are two different and well-described mouse inflammation models [7], [8]. To elucidate how RAGE is involved in the PC activation process we measured RAGE dependent aPC levels and EPCR and TM protein- and mRNA expression. Furthermore, we analyzed RAGE dependent MAPK (mitogen activated protein kinase) activation and endothelial ICAM-1 and VCAM-1 expression in response to PC treatment.

## Materials and Methods

### Animals

C57BL/6J mice (male) were purchased from Charles River (Sulzfeld, Germany). *RAGE^−/−^* mice (male) were generated as described earlier and backcrossed for at least 15 generations into C57BL/6J background [25]. All mice were maintained at a 12 hour light/dark cycle with ad libitum access to food and water at the Central Animal Facility of the University of Heidelberg, Germany. For all experiments, mice were at least 8 weeks of age. All animal experiments were conducted to the German guidelines for animal care and were approved by the Animal Care and Use Committee of the Regierungspraesidium Karlsruhe, Germany (AZ 35-9185.81/G85/11).

### Protein C, Cytokines, and Special Reagents

Human protein C concentrate CEPROTIN [Protein C Concentrate (Human)] was kindly provided from Baxter (Unterschleissheim, Germany), dissolved as indicated in the drug data sheet to an isotonic working solution of 100 U/ml protein C (1 U = 4 µg PC). PC solution was further dissolved in normal saline to 200 µl and intravenously administered. In all experiments, PC was administered at 100 U/kg (referring equivalent to 400 µg/kg) 3 h before intravitalmicroscopic observation, or as indicated. Human activated protein C (Enzyme Research Laboratory, Swansea, UK) was diluted in normal saline to a working solution of 100 µg/ml and was systemically injected into mice at 24 µg/kg/h, 3 h before intravitalmicroscopic observation, or as indicated. APC was added to cultured murine aortic endothelial cells, as indicated. In designated *in vivo* experiments, recombinant murine TNFα (R&D Systems, Wiesbaden, Germany) was applied intrascrotally at 500 ng per mouse for 3 h.

### Coagulation Assays

To investigate the coagulation parameters during PC therapy, mice were first anesthetized by intraperitoneal (i.p.) injection of ketamine (125 mg/kg body weight, Pfizer, Karlsruhe, Germany) and xylazine (12.5 mg/kg body weight, Alverta, Neumuenster, Germany). Then, blood was taken as final blood sample by heart puncture 3 h after application of PC or saline in TNFα induced inflammation. Using citrated plasma samples, INR (international normalized ratio), aPTT (activated Partial Thromboplastin Time), fibrinogen and protein C levels were measured by the laboratory core facility of the Dept. of Clinical Chemistry, University of Heidelberg, Germany. For INR, aPTT and fibrinogen standard assay was performed. Levels of zymogen protein C were measured photometrically using a chromogenic substrate (PCa, American Diagnostica, Greenwich, Connecticut, USA) crossreacting with human and murine protein C.

Activation of human protein C was analyzed as previously described [33], with some modifications. Briefly, mice were injected with 100 U/kg of human protein C into the tail vein. As positive controls, in some experiments 50 milliunits of human α-thrombin (Hemochrom Diagnostica, Essen, Germany) were additionally injected 10 minutes before blood sampling. In another set of experiments, animals were continuously injected with activated protein C (Enzyme Research Laboratories, Swansea, UK) at 24 µg/kg/h. Saline injected WT and *RAGE^−/−^* mice served as negative controls. 30 minutes after PC, aPC or saline administration, blood was taken as a final blood sample by heart puncture into 0.38% sodium citrate and 50 mM benzamidin HCl. Human activated protein C was captured from these plasma samples using the HAPC1555 antibody (kindly provided by C. T. Esmon, Oklahoma Medical Research Foundation, Oklahoma City, USA), which is an highly specific mouse monoclonal antibody against human aPC, developed by standard techniques [34]. Because of the antibodies capacity for capturing from plasma, the direct detection of aPC plasma-concentrations is possible [35]. The activity of the captured human PC was determined using a chromogenic substrate (PCa, American Diagnostic) [12], [36], [37].

### Intravital Microscopy

As recently reported, we used the cremaster muscle models of trauma- and TNFα- induced inflammation [8]. Briefly, after intraperitoneal anesthesia (as mentioned above), mice were placed on a heating pad to maintain body temperature. Intravital microscopy was conducted on an upright microscope (Leica, Wetzlar, Germany) with a saline immersion objective (SW40/0.75 numerical aperture, Zeiss, Jena, Germany).

### Cremaster Muscle Preparation

The surgical preparation of the cremaster muscle was conducted as described previously (trauma-induced inflammation) [8]. Briefly, the scrotum was opened and the cremaster muscle exteriorized. After longitudinal incision and spreading of the muscle over a cover glass, the epididymis and testis were mobilized and pinned aside leading to full microscopic access to the cremaster muscle microcirculation. Microscopic observation of cremaster muscle venules of 20–40 µm diameters were recorded via CCD camera (CF8/1, Kappa, Gleichen, Germany) on a Panasonic S-VHS recorder. The cremaster muscle was superfused with thermo-controlled (35°C) bicarbonate-buffered saline. The number of adherent leukocytes (firm adhesion for >30 s) was assessed as adherent cells per mm^2^ vessel surface area [8]. In certain experiments, mice were injected with 500 ng recombinant murine TNFα intrascrotally 3 h before intravital microscopy (TNFα-induced inflammation).

In a separate set of experiments, cremaster muscle whole mounts were obtained as described before [8], and analyzed for extravascular leukocytes after fixation and Giemsa staining using a Leica DMRB upright microscope and a ×63/0.75NA oil immersion objective (both Leica, Wetzlar, Germany).

After the respective experiment, anesthetized mice were killed by cervical dislocation.

### Cell Culture

For several of the following in vitro experiments cultured murine aortic endothelial cells (MAECs) of WT and *RAGE^−/−^* mice were used. Endothelial cells were isolated and cultured as described previously [38]. Briefly, 3-mm long freshly harvested and cleaned aortic rings were seeded into Matrigel-coated culture dishes (BD, San Jose, CA, USA) and incubated at 37°C, 5% CO_2_ in Dulbecco’s Modified Eagle Medium (CCpro, Oberdorla, Germany), supplemented with 10% heat inactivated bovine serum (PAA, Cölbe, Germany), 1% Pen/Strep and 1% non-essential amino-acids (both CCpro).

### *In vitro* PC Activation

To investigate the activation of human PC *in vitro*, WT and *RAGE^−/−^* MAECs were grown to near confluence in 24-well plates (Greiner, Frickenhausen, Germany). Cells were incubated with human PC (12,5 µg/ml, CEPROTIN®, Baxter, Unterschleissheim, Germany) and α-thrombin (0,25 U/ml, Hemochrom Diagnostica, Essen, Germany) for 1 hour at 37°C. Then, antithrombin III (40 U/ml, Kybernin P, CSL Behring, Marburg, Germany) and hirudin (400 U/ml, Sigma, Taufkirchen, Germany) were added. Saline incubation for 1 hour at 37°C served as negative control. Concentration of activated protein C in supernatants was determined using a chromogenic substrate (S-2366, Hemochrom Diagnostica, Essen, Germany).

### Flow Cytometry

For investigation of EPCR, TM, ICAM-1 and VCAM-1 expression WT and *RAGE^−/−^* MAECs were grown to near confluence in 6-well plates (Greiner, Frickenhausen, Germany), then incubated with TNFα at 25 ng/ml for 4 h, harvested with Accutase (PAA, Cölbe, Germany) and incubated in the dark for 45 min on ice with a PE-conjugated anti-CD 201 (EPCR, clone RCR-16, eBioscience, San Diego, USA), PE-conjugated anti-TM (R & D Systems, Minneapolis, USA), PE-conjugated anti-ICAM-1 mAB (clone YN1/1.7.4 eBioscience, San Diego, USA), anti-mouse VCAM-1 mAb (clone 429 MVCAM.A BioLegend, San Diego, USA) or respective isotype control antibody (eBioscience, San Diego, USA and BD ). Unstimulated cells served as controls. In certain experiments cells were pretreated with aPC (10 µg/ml for 16 h).

To analyse NF-κB p65 (Ser536)-, p38 MAPK (Thr180/Tyr182)- and p44/42 MAPK (Erk1/2: Thr202/Tyr204)- phosphorylation, WT and *RAGE^−/−^* MAECs were pretreated with aPC (10 µg/ml, 20 min) before TNFα-stimulation (100 ng/ml, 15 min). After fixation (4% PFA) and permeabilization (0.01% Triton X-100, Sigma, Taufkirchen, Germany), cells incubated in the dark for 45 min at 4°C with PE-conjugated rabbit anti-Phospho -p65, -p38 or -p44/42 mAB or respective isotype control antibody (Cell Signaling Technologies, Danvers, USA). TNFα-stimulated cells without aPC treatment served as treatment controls, while prepared cells without TNFα served as preparation controls. All flow cytometric analyses were performed using the four-decade FACS Scan LSRII with DIVA software package (Becton Dickinson, San Jose, USA).

### Immunohistochemistry

To investigate the effect of aPC on ICAM-1 and VCAM-1 expresssion in TNFα-stimulated cremaster muscle venules, immunohistochemical analysis of whole mount cremaster muscles was performed as described [14], [39]. Briefly, primary antibodies against murine ICAM-1 (YN1, monoclonal rat anti-mouse, 30 µg/mouse, eBioscience, San Diego, USA) and VCAM-1 (MVCAM.A 429, 30 µg/mouse, Abd Serotec, Oxford, UK) were systemically injected in the carotid artery and incubated for 10 minutes. Because of the intravascular antibody application after exteriorization of the cremaster muscle, binding of antibodies is mostly restricted to surface expressed antigens within the vasculature. Surgically prepared cremaster muscle whole mounts were transferred onto adhesive slides (Superfrost, Menzel, Braunschweig, Germany) and fixed overnight in acetone at −18°C. The tissue was incubated with a biotin-conjugated goat antibody directed against rat immunoglobulin G (Southern Biotech, Birmingham, Alabama, USA) and stained for endothelial ICAM-1 and VCAM-1 expression using 3,3′-diaminobenzidine (Vector Laboratories, Burlingame, USA). APC or saline were administered as in the above described *in vivo* experiments.

### RNA Isolation, Reverse Transcription, and Real-time Quantitative Polymerase Chain Reaction

Total RNA of TNFα-stimulated WT and *RAGE^−/−^* MAECs was extracted by TriFast (Peqlab, Erlangen, Germany) and treated with DNAse I (Sigma-Aldrich, Taufkirchen, Germany) to digest genomic DNA. RNA was transcribed to complementary DNA (cDNA) by using Moloney murine leukemia virus reverse transcriptase, random primers (both from Promega, Mannheim, Germany) and specific oligo(dT)primers (Carl Roth, Karlsruhe, Germany). Relative mRNA transcript levels were analyzed with a LightCycler (Roche Applied Science, Mannheim, Germany) and a respective FastStart DNA Master Hybridization Probes kit using the TaqMan method. ALAS (Aminolevulinate Synthase) served as housekeeping gene. The specific primers (see Table 1) and probes were designed using the Universal Probe Library Assay Design Center (Roche Applied Science). Primers (see Table 1) were synthesised at TIB MOLBIOL (Berlin, Germany), probes (# 26 for EPCR, #81 for TM and #40 for ALAS) at Roche (Mannheim, Germany).

**Table 1:** Primer Sequences.

Primer	Sequence
**Endothelial protein C receptor (EPCR)**	
forward	5′- agcgcaaggagaacgtgt -3′
reverse	5′ - gggttcagagccctcctc -3′
**Thrombomodulin (TM)**	
forward	5′ - atgcgtggagcatgagtg -3′
reverse	5′ - ctggcatcgaggaaggtc -3′
**Aminolevulinate Synthase (ALAS)**	
forward	5′ - ccctccagccaatgagaa -3′
reverse	5′ - gtgccatctgggactcgt -3′

### Statistics

All statistical analyses were performed using Prism 4 (GraphPad, La Jolla, USA). Statistical significance between groups and treatments were compared with one-way ANOVA followed by a multiple pairwise comparison test or by Student’s t-Test. Statistical significance was set at *P*<0.05.

## Results

### Impact of PC and aPC on Leukocyte Adhesion and Transmigration in WT Mice

The capacity of PC and aPC to inhibit leukocyte recruitment in wild type (WT) mice was observed by intravital microscopy of leukocyte adhesion in postcapillary venules of inflamed cremaster muscles in two established models. While in the trauma-model the inflammatory response results from the surgical preparation of the mouse cremaster muscle, the TNFα-induced inflammation is caused by intrascrotally injection of TNFα [7], [8]. In line with recent studies, we found that the majority of recruited leukocytes in these inflammation models are neutrophils (about 85%, not depicted) [8], [40]–[43]. Microvascular and hemodynamic parameters did not vary significantly between the treatment groups and genotypes (Supplemental Table S1).

**Figure 1:**
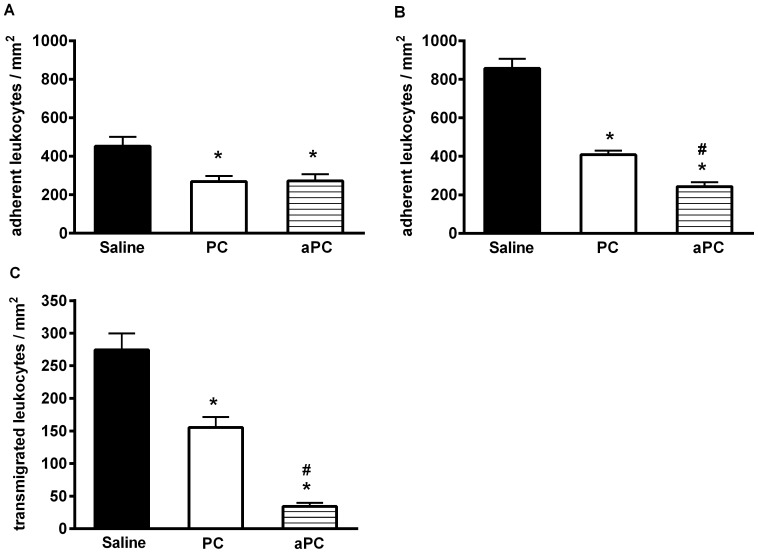
Effect of PC and aPC on leukocyte adhesion and transmigration in WT mice. Leukocyte adhesion (number of adherent cells per mm^2^ of surface area) in cremaster muscle venules of wild-type (WT) mice, treated with and without PC (100 U/kg, 3 h) or aPC (24 µg/kg/h for 3 h) during trauma (A) and TNFα (B) induced inflammation was investigated via intravital microscopy. Leukocyte transmigration (number of transmigrated leukocytes per mm^2^ surface area) was analyzed in Giemsa-stained cremaster muscle whole mounts in the TNFα model in WT mice with and without PC (100 U/kg, 3 h) or aPC (24 µg/kg/h, 3 h) treatment (C) obtained after the respective intravital microscopic experiment. All values are presented as mean+SEM from three or more mice per group. Significant differences (*P*<0.05) to saline treated WT (control) and PC or aPC-treated WT mice are indicated by the asterisks and pound keys respectively.

Dose finding and timing studies in the TNFα-model (Figure S1A–C) revealed that treatment with 100 U PC/kg or 24 µg aPC/kg/h, 3 hours before microscopic observation, very effectively reduced the number of adherent leukocytes. Thus, all further *in vivo* experiments were performed by using these PC and aPC doses for a 3 hour treatment. During trauma-induced inflammation (Figure 1A) and after TNFα-stimulation (Figure 1B, see also supplemental Movie S1) leukocyte adhesion was significantly decreased in PC-treated mice compared to saline treated control mice. Notably, treatment with aPC blocked leukocyte adhesion (see also supplemental Movie S2) and showed even enhanced effects when compared to PC during TNFα-induced inflammation (Figure 1B).

In order to investigate if PC-induced inhibition of leukocyte adhesion also has an impact on transmigration, we performed Giemsa-staining of TNFα-stimulated cremaster muscle whole mounts, obtained after the respective intravital microscopic experiment (Figure 1C and Figure S3A, C & E). Similar to leukocyte adhesion, leukocyte transmigration was significantly reduced by treatment with PC and aPC, suggesting that the anti-inflammatory properties of PC/aPC on leukocyte adhesion translate into transmigration.

The results of supplemental Table S2 confirm that the injected PC significantly increased plasma PC levels, while basic plasmatic coagulation parameters were not altered (aPC levels were investigated later in this study).

### Role of RAGE for PC and aPC Induced Inhibition of Leukocyte Adhesion and Transmigration

**Figure 2:**
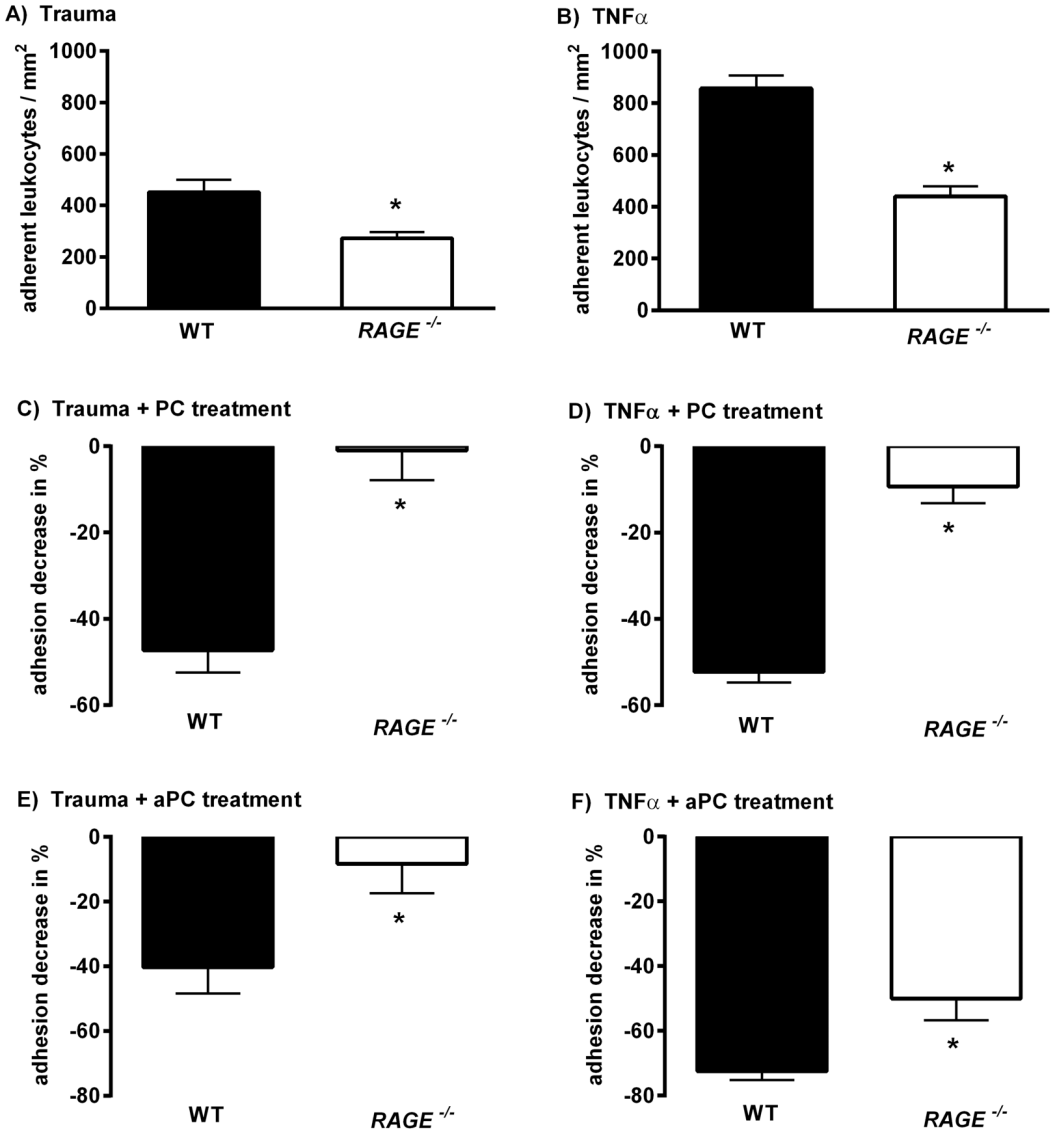
Effect of PC and aPC on leukocyte adhesion in *RAGE^−/−^* mice compared to WT control mice. Comparison of intravital microscopic data of leukocyte adhesion (number of adherent cells per mm^2^) during trauma-induced inflammation (A) and in TNFα-stimulated (B) cremaster muscle venules of saline treated WT (control) and *RAGE^−/−^* mice. Effect of PC treatment (100 U/kg, 3 h) on leukocyte adhesion in WT and *RAGE^−/−^* mice represented as relative decrease [%] of leukocyte adhesion during trauma (C) and TNFα (D) induced inflammation. Effect of aPC treatment (24 µg/kg/h, 3 h) on leukocyte adhesion in WT and *RAGE^−/−^* mice represented as relative decrease [%] of leukocyte adhesion during trauma (E) and TNFα (F) induced inflammation. All values are presented as mean+SEM from three or more mice per group. Significant differences (*P*<0.05) to WT mice are indicated by the asterisks.

To elucidate the role of RAGE for mediation of anti-inflammatory properties of PC and aPC, leukocyte adhesion was observed during trauma- and TNFα-induced inflammation via intravital microscopy in WT and *RAGE^−/−^* mice under control conditions (saline control) or after PC/aPC treatment. As previously reported [7], [8] *RAGE^−/−^* mice showed a significantly reduced number of adherent cells in inflamed cremaster muscle venules compared to WT mice (Figure 2A & B and supplemental Movies S1 & S3, respectively). Figure 2C - F depicts the anti-inflammatory effect of PC/aPC as relative inhibition of leukocyte adhesion (%). Both aPC and PC efficiently blocked leukocyte adhesion in WT mice during trauma-induced inflammation (by almost 50%), whereas in *RAGE^−/−^* mice leukocyte adhesion was neither influenced by PC nor by aPC (Figure 2C & E). During TNFα-stimulation PC exerted a profound anti-inflammatory effect in WT mice (about 50%), but not in *RAGE^−/−^* mice (Figure 2D). Notably, in contrast to PC, aPC treatment strongly blocked leukocyte adhesion in both WT and *RAGE^−/−^* mice in the TNFα-model (supplemental Movies S2 & S4, respectively), although the inhibitory capacity was more pronounced in WT than in *RAGE^−/−^* mice (70% vs. 50%, Figure 2 F and supplemental Figure S4).

Moreover, the PC- and aPC-induced inhibition of leukocyte adhesion did nicely translate into leukocyte transmigration as seen in Giemsa-stained TNFα-stimulated cremaster muscle whole mounts obtained after respective intravital microscopic experiment (Figure S2A & B and supplemental Figure S3A–F). These results suggest a role of RAGE for mediation of PC- and, in part, aPC-induced inhibition of leukocyte adhesion and transmigration. The fact that PC was ineffective and aPC partially effective in *RAGE^−/−^* mice, depending on the inflammatory stimulus, raised the question whether RAGE might be involved in the activation process of PC. Precisely, these data indicate that RAGE is required for PC-activation following stimulation with TNFα.

### Role of RAGE for Activation of PC

To investigate the role of RAGE in PC activation, aPC plasma concentrations were measured in PC- and aPC-treated WT and *RAGE^−/−^* mice and compared to respective saline treated control mice (negative controls) and PC/thrombin-co-injected WT mice (positive controls).

**Figure 3:**
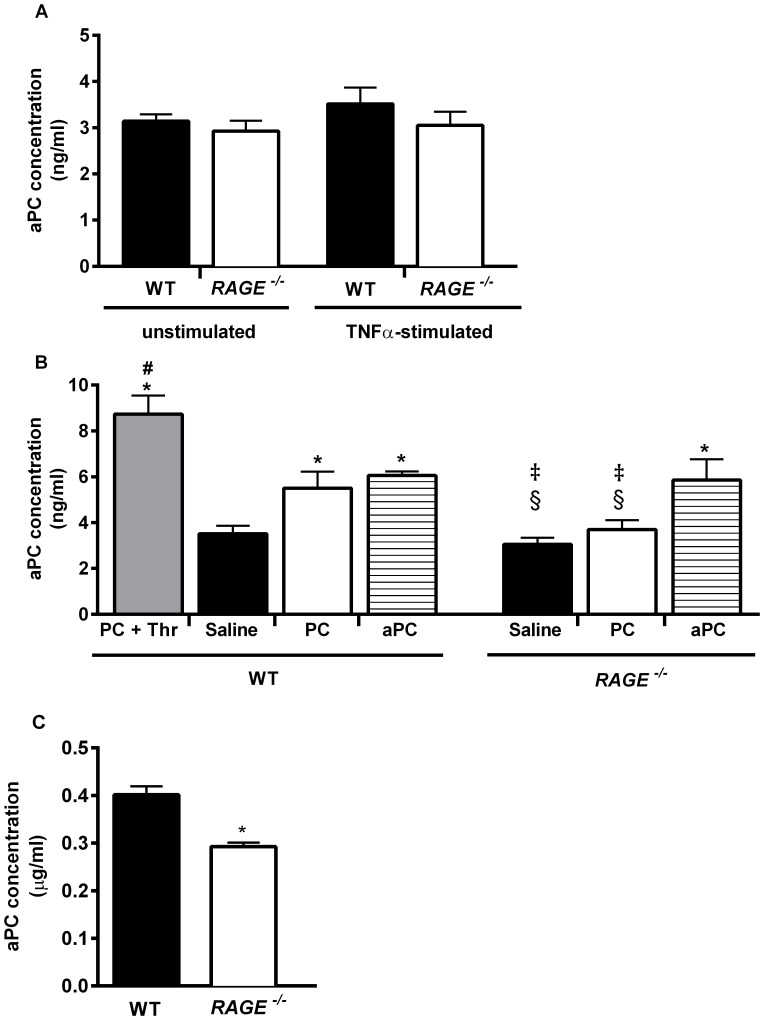
*In vivo* and *in vitro* PC activation. Basal plasma concentration of endogenous activated protein C was analyzed in unstimulated and TNFα-stimulated WT and *RAGE^−/−^* mice (A, without PC or aPC application). TNFα-stimulated WT and *RAGE^−/−^* mice were treated with 100 U/kg PC or 24 µg/kg/h aPC for 30 min and compared to saline treated control mice (B). In some experiments, additionally administered human α-thrombin in WT mice enhanced PC activation and served as positive control (PC+Thr). APC concentration of WT and *RAGE^−/−^* murine aortic endothelial cells was assessed after treatment with PC (12,5 µg/ml) and α-thrombin (0,25 U/ml) for 1 hour and adjusted to negative controls (C). All values are presented as mean+SEM from at least three mice or separate experiments per group (A & B). (C). Significant differences (*P*<0.05) in (A) are indicated to saline treated WT control mice (*) and, to WT mice treated with PC and aPC (§). aPC-treated *RAGE^−/−^* mice (‡) are significant to PC and saline treated control *RAGE^−/−^* mice. PC+Thr. treated WT mice are significant to all other groups (#). Significant differences (*P*<0.05) in (C) are indicated to WT cells.

Basal PC and aPC levels were similar between WT and *RAGE^−/−^* mice (Table S2 and Figure 3A). Moreover, endogenous aPC levels did not vary between unstimulated and TNFα-stimulated mice (Figure 3A) and therefore the following experiments were performed in TNFα-stimulated mice only. As expected, maximal activation was achieved by co-injection of PC with thrombin in positive controls. APC plasma concentration significantly increased (to comparable levels as after aPC-treatment) 30 minutes after PC injection in WT mice, indicating a sufficient activation of PC. However, in PC-treated *RAGE*-deficient mice aPC plasma concentration did not significantly differ from *RAGE^−/−^* control mice (Figure 3B). To investigate the role of endothelial RAGE for PC activation we performed an *in vitro* PC activation assay with PC and thrombin treated and untreated WT and *RAGE^−/−^* endothelial cells. As depicted in Figure 3C, *in vitro* PC activation was significantly reduced in *RAGE^−/−^* endothelium compared to WT endothelium. These data support the hypothesis that PC activation is impaired in the absence of endothelial RAGE.

### RAGE Dependent Endothelial Expression of EPCR and TM

**Figure 4:**
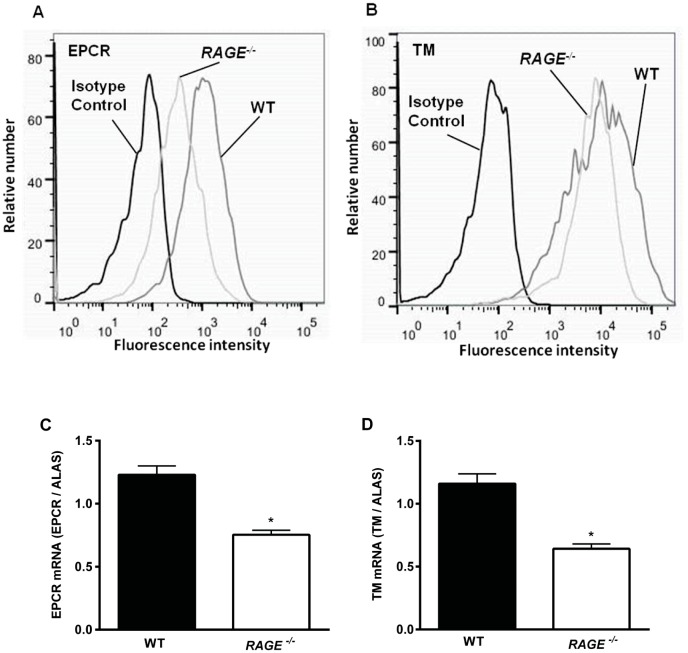
Endothelial EPCR and TM protein- and mRNA expression *in vitro*. EPCR (A) and TM (B) protein-expression was measured in cultured WT and *RAGE^−/−^* murine aortic endothelial cells (MAECs) after stimulation with TNFα for four hours. Surface expression was compared to isotype control. Representative histograms are shown for three separate experiments. mRNA-Expression of EPCR (C) and TM (D) was analyzed of cytokine stimulated (TNFα 25 ng/ml, 4 h) WT and *RAGE^−/−^* MAECs in relation to housekeeping gene mRNA expression (ALAS) in three separate experiments. Significant differences (*P*<0.05) to WT control cells are indicated by the asterisks.

To elucidate the mechanisms of RAGE-dependent protein C activation, FACS analysis of endothelial expression of EPCR and TM were performed in TNFα-stimulated WT and *RAGE^−/−^* murine aortic endothelial cells (MAECs). While WT MAECs strongly express EPCR and TM, *RAGE^−/−^* MAECs showed a lower EPCR and - less attenuated - TM expression (Figure 4A & B). These data were supported by analysis of EPCR- and TM mRNA expression (Figure 4C & D). The mRNA-expression of both molecules was significantly reduced in *RAGE^−/−^* MAECs compared to expression in WT cells. Since EPCR triggers TM mediated PC activation [44], [45], this finding might – at least in part - explain the insufficient PC activation in the absence of RAGE.

### Role of RAGE for PC-induced Inhibition of MAPK Activation

**Figure 5:**
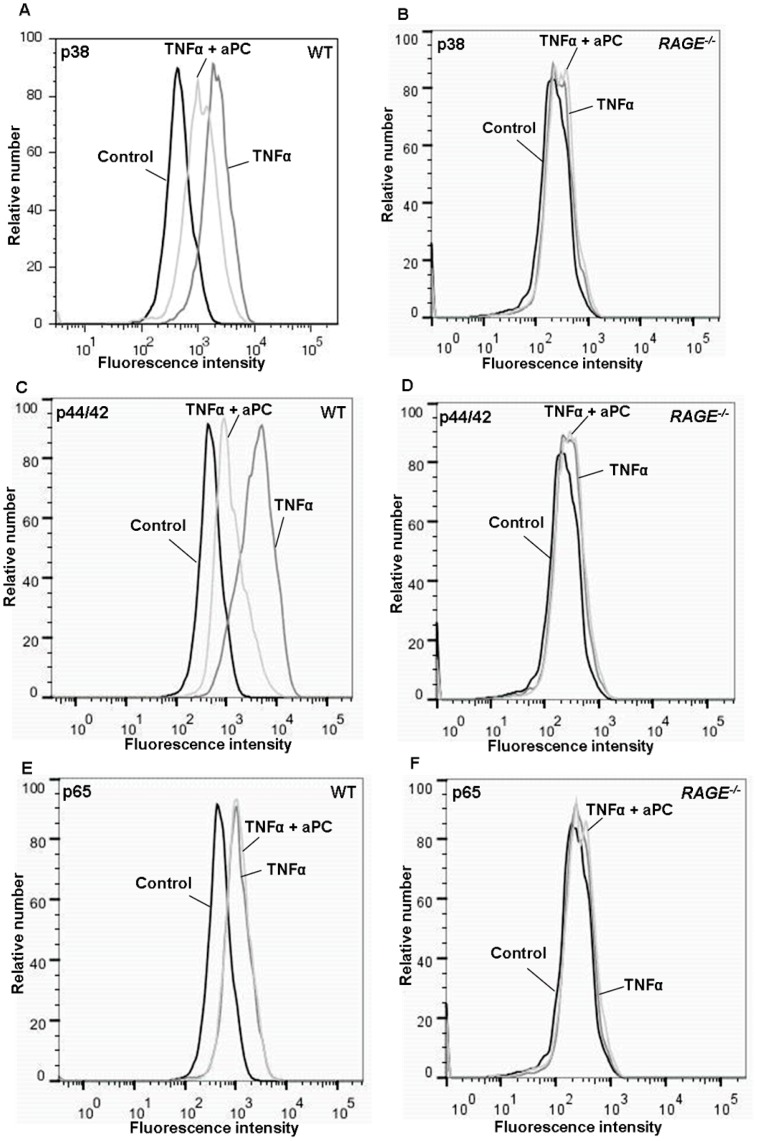
Effect of aPC on activation of intracellular signalling pathways *in vitro*. Activation of p38 MAPK, p44/42 MAPK and NF-κB (p65) of cultured WT (A, C & E) and *RAGE^−/−^* (B, D & F) endothelial cells. Phosphorylation of p38 MAPK, p44 MAPK and NF-κB (p65) was measured after 15 min TNFα stimulation (100 ng/ml) with and without 20 min aPC pre-incubation (10 µg/ml) and was compared to prepared cells without TNFα stimulation (controls).

The next step was the investigation of the intracellular signalling linking RAGE with the PC pathway. Since aPC is capable to diminish MAPK activation [46], the aPC effect on TNFα-induced phosphorylation of p38 MAPK and p44/42 MAPK and p65 (NF-κB) in *RAGE^−/−^* MAECs was compared to WT MAECs and to respective controls (without TNFα-stimulation and isotype controls). To mention, the preparation and harvesting procedure of the cells reflects best the surgical preparation during the trauma-induced inflammation *in vivo* model. P38, p44/42 and p65 phosphorylation of these preparation controls (referred to as control in Figure 5) did not differ from the respective isotype controls *RAGE^−/−^* and WT MAECs (not depicted), suggesting that there was no MAPK or NF-κB activation upon cell preparation.

In line with Guitton *et al.* [46], aPC reduced TNFα-induced phosphorylation of p38 MAPK (Figure 5A) and p44/42 MAPK (Figure 5C) in WT cells. In contrast, there was no p38 MAPK (Figure 5B) and p44/42 MAPK (Figure 5D) activation in *RAGE^−/−^* cells and consequently no respective aPC effect. However, aPC did not affect p65 phosphorylation, neither in WT (Figure 5E) nor in *RAGE^−/−^* (Figure 5F) cells. Noteworthy, only intracellular phosphorylation of p38, p44/42 and p65 were measured by our means, not total contents. Nevertheless, these results indicate that RAGE plays a role in MAPK mediated aPC signalling.

### Role of RAGE for PC-induced Downregulation of ICAM-1 and VCAM-1

**Figure 6:**
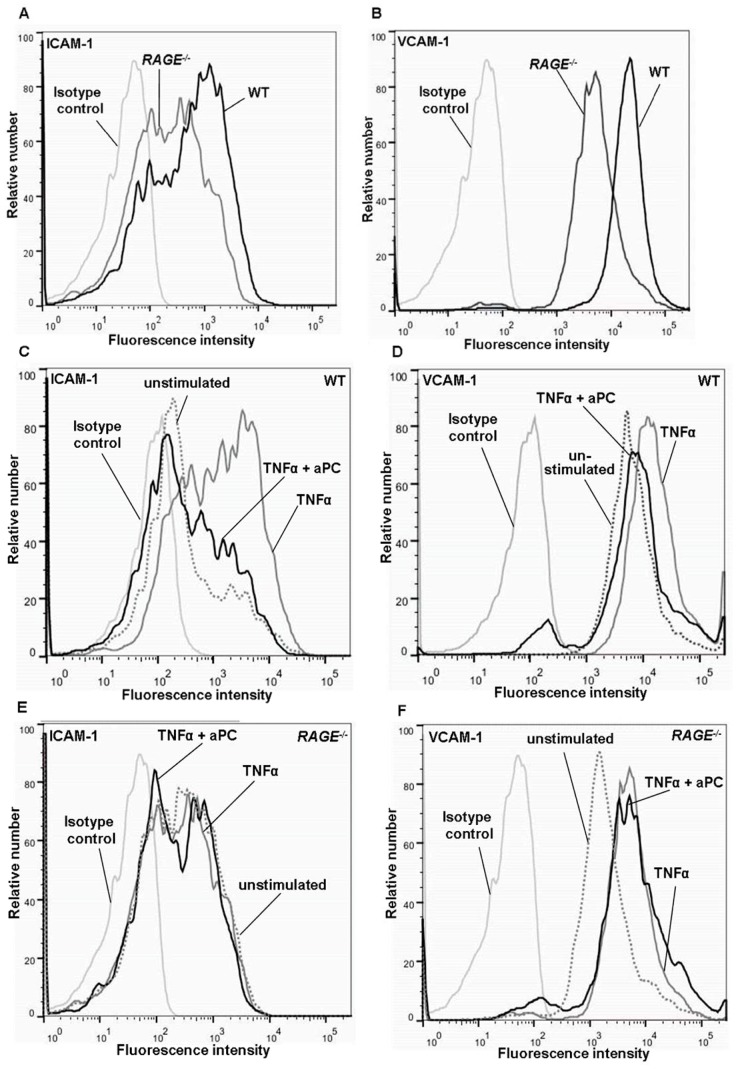
Effect of aPC on endothelial ICAM-1 and VCAM-1 expression *in vitro*. ICAM-1 (A) and VCAM-1 (B) expression of cultured WT and *RAGE^−/−^* endothelial cells was measured after stimulation with TNFα for four hours (25 ng/ml). ICAM-1 and VCAM-1 expression of WT (C and D respectively) and *RAGE^−/−^* (E and F respectively) endothelial cells was then assessed after TNFα stimulation with and without aPC pre-incubation (10 µg/ml 16 h before TNFα) and compared to respective isotype and unstimulated controls. Representative histograms are shown for three separate experiments.

**Figure 7:**
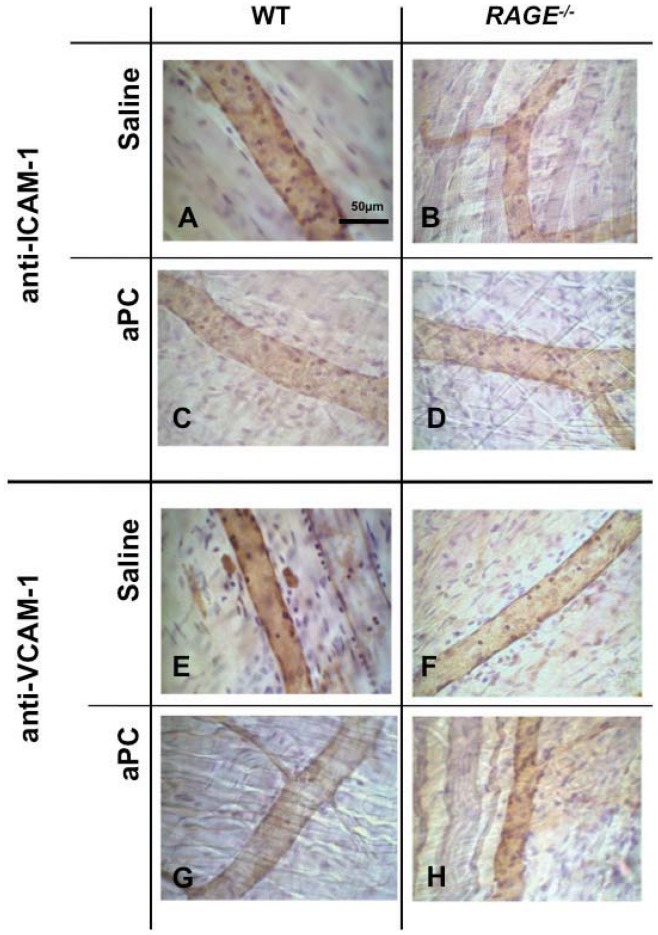
Effect of aPC on endothelial ICAM-1 and VCAM-1 expression *in vivo*. *In vivo* endothelial immunostaining in WT (left side) and *RAGE^−/−^* (right side) mice after treatment with aPC (24 µg/kg/h, 3 hours; C & D and G & H) or saline (A & B and E & F) was carried out to receive further information about ICAM-1 (A–D) and VCAM-1 (E–H) expression in TNFα-stimulated cremaster muscle venules. Representative micrographs are shown for at least three mice per group. Reference bar for (A–H) is shown in (A) and represents 50 µm.

We next addressed the question whether RAGE is involved in PC-dependent regulation of effector molecules of leukocyte recruitment, like expression of leukocyte adhesion molecules ICAM-1 and VCAM-1. Therefore, ICAM-1 and VCAM-1 expression on MAECs was assessed by flow cytometry and by *in vivo* immunohistochemistry in the presence and absence of RAGE. First, TNFα-induced expression of ICAM-1 (Figure 6A) and VCAM-1 (Figure 6B) of *RAGE^−/−^* MAECs was compared with WT endothelial cells, showing a reduced expression of both adhesion molecules on *RAGE^−/−^* cells. Next, we demonstrated that aPC is capable to downregulate TNFα−induced endothelial ICAM-1 (Figure 6C) and VCAM-1 (Figure 6D) expression under WT conditions. Since VCAM-1 (Figure 6F) and particularly ICAM-1 (Figure 6E) expression is hardly stimulated by TNFα in the absence of RAGE, the capacity of aPC to block TNFα-induced upregulation of these adhesion molecules is difficult to measure in this model. At least, these data indicate that aPC is not able to downregulate VCAM-1 and ICAM-1 further than constitutional baseline expression levels (unstimulated) in *RAGE^−/−^* cells. To step further, the *in vivo* situation was investigated by immunohistochemistry of ICAM-1 and VCAM-1 in TNFα-stimulated cremaster muscles of WT and *RAGE^−/−^* mice (Figure 7A–H). Similar to flow cytometric analysis, endothelial ICAM-1 and VCAM-1 expression in *RAGE* deficient mice (Figure 7B & F) were lower than in WT mice (Figure 7A & E). While aPC treatment reduced ICAM-1 (Figure 7C) and VCAM-1 (Figure 7G) on WT endothelium, there was no such effect on *RAGE^−/−^* endothelium (Figure 7B & H). These findings suggest that both RAGE and PC are involved in the regulation of endothelial ICAM-1 and VCAM-1 and that RAGE might be at least in part important for PC- induced downregulation of these adhesion molecules.

## Discussion

This study is the first that provides evidence that RAGE is involved in mediation of anti-inflammatory properties of PC and that it supports PC activation in a model-dependent manner.

To dissect how the anti-inflammatory PC pathway is related to RAGE, the capacity of PC and aPC to block leukocyte adhesion and transmigration was investigated in *RAGE^−/−^* and WT mice using two different established murine cremaster muscle inflammation models, trauma- and TNFα-induced inflammation, provoking mainly neutrophil infiltration [7], [8], [14], [42], [43]. While there was a strong anti-inflammatory effect of PC and aPC in WT mice during trauma-induced inflammation, both treatments failed in *RAGE^−/−^* mice in that model supporting the hypothesis that PC and aPC might require RAGE in order to inhibit leukocyte adhesion.

It is known that in the *in vivo* inflammation model of 3 hours TNFα-stimulation RAGE and its signalling properties are crucial for mediation of leukocyte recruitment [7], [8]. In this model, the strong inhibition of leukocyte adhesion after PC and aPC treatment in WT mice is contrasted by the lacking effect of PC in the absence of RAGE. However, aPC treatment reduced leukocyte adhesion in *RAGE^−/−^* mice, indicating that RAGE may be linked to PC activation. This hypothesis was supported by the in vivo aPC capture assay which showed high plasma aPC levels after zymogen PC injection in WT mice, but significantly lower plasma aPC levels in *RAGE^−/−^* mice. The insufficient *in vitro* PC activation of *RAGE^−/−^* endothelial cells underlines that, in particular, endothelial RAGE is essential for PC activation. Nevertheless, based on our *in vitro* results we cannot exclude a role of leukocyte-expressed RAGE for PC activation *in vivo.* Exploring underlying mechanisms, we observed reduced endothelial surface and mRNA expression of EPCR and TM in *RAGE-*deficient endothelium. While TM is the main cofactor for PC activation [47], [48], EPCR is known to be critical for mediating anti-inflammatory functions of aPC by cleaving PAR1 which results in intracellular signalling [45], [49], [50]. Interestingly, Bae *et al*. showed that EPCR, TM and PAR1 have to be colocalized in membrane lipid rafts of endothelial cells for effective PC activation and intracellular aPC signalling [51]. As a consequence, the reduced expression of TM and EPCR in *RAGE^−/−^* mice might be responsible for both, insufficient PC activation on the one hand and impaired anti-inflammatory PC signalling on the other hand. Furthermore, one possible mechanistic link between RAGE and TM and EPCR could be a RAGE regulated transcription of TM and EPCR by specificity protein 1 (Sp1) transcription factor binding sites which are located in the promoter regions of all the three molecules [52]–[54].

Interestingly, the efficacy of aPC in *RAGE* deficient mice seems to be dependent on the kind of inflammatory stimulation since aPC failed to block leukocyte recruitment during short-term stimulation in the trauma model, whereas it was effective during long-term pro-inflammatory stimulation with TNFα in these mice. An explanation of this phenomenon could be that the formation of lipid rafts and clusters containing PAR1 may increase by stronger inflammatory stimulation or that aPC might rather use other receptors than the common PC pathway molecules under these conditions (like Shingosine-1-phosphate receptor 1 [55], the angiopoietin Ang/Tie 2 axis [56] and PAR 3 [4]). Alternatively, depending on the inflammatory stimulus, different cell types might be involved in the RAGE dependent aPC signalling.

Next, we aimed to link RAGE and PC pathway signalling more downstream up to effector molecules of leukocyte adhesion. As recently discovered, the intracellular protein C signalling of endothelial cells involves NF-κB and ERK1/2 MAPK which may in turn regulate endothelial expression of adhesion molecules [46]. Therefore, we investigated the impact of aPC on activation of p38 and p44/42 (ERK 1/2) mitogen-activated protein kinases (MAPK) and NF-κB, as well as endothelial expression of ICAM-1 and VCAM-1 upon cytokine stimulation. In line with Guitton *et al*. [46], we found that aPC reduced phosphorylation of p38 and p44/42 (ERK 1/2) mitogen-activated protein kinases in TNFα-stimulated WT MAECs, which was not the case in *RAGE^−/−^* cells. Subsequently, aPC down-regulates endothelial ICAM-1 and VCAM-1 expression in WT endothelial cells but not in *RAGE^−/−^* endothelial cells. These data are in contrast to Uchiba *et al*. showing that the MAPK pathway is activated by aPC [57]. However, their experimental setting (i.e. cell types, kind and time of pro-inflammatory stimulation) was different from our study.

Noteworthy, the known anti-inflammatory phenotype of *RAGE* ^−/−^ mice [25] reflected by impaired inflammatory signalling, downregulated ICAM-1 and VCAM-1 and reduced leukocyte adhesion and transmigration hamper strong conclusions about anti-inflammatory effects of aPC in the absence of RAGE.

In addition, we cannot exclude that PC interferes with the interaction of RAGE with its ligands, which has been proposed for HMGB1 [58]–[60] and Mac-1 [61]. In this regard, Fink *et al.* showed that soluble EPCR mediates monocyte adhesion by direct binding to Mac-1, an interaction which might possibly involve RAGE too [62]. Notably, and in contrast to the study of Fink *et al*., the majority of recruited leukocytes in our experimental inflammation models are neutrophils [8], [40]–[43].

Another limitation of the study is that it is not able to clearly dissect the contribution of leukocyte expressed RAGE from endothelial RAGE. This, however, is beyond the scope of this article and should be performed in future studies.

Taken together, our findings suggest that RAGE mediates PC-induced anti-inflammatory properties and that PC activation is dependent on RAGE potentially involving TM and EPCR. Thus, our study may offer new perspectives for the development of novel anti-inflammatory strategies.

## Supporting Information

Figure S1 **Dose dependent impact of PC (A) on leukocyte adhesion (number of adherent cells per mm^2^ of surface area) in TNFα (3 h) inflamed cremaster muscle venules of WT mice.** Time dependent effect of protein C (PC 100 U/kg; B) and activated protein C (aPC 24 µg/kg/h; C) treatment for leukocyte adhesion in cremaster muscle venules of WT mice were measured after 3 hours TNFα stimulation. All leukocyte adhesion values were obtained by intravital microscopy and are presented as mean+SEM from three or more mice per group. Significant differences (*P*<0.05) to control mice are indicated by the asterisks.
(TIF)

Figure S2 **Effect of PC and aPC on leukocyte transmigration in giemsa-stained cremaster muscle whole mounts of** ***RAGE^−/−^*** **mice compared to WT control mice.** Cremaster muscle whole mounts were obtained after the respective intravital microscopic experiment followed by giemsa-staining. Comparison of relative decrease of leukocyte transmigration [%] during TNFα induced inflammation of PC (100 U/kg, 3 h) (A) and aPC (24 µg/kg/h, 3 h) (B) treated WT and RAGE−/− mice. All values are presented as mean+SEM from three or more mice per group. Significant differences (P<0,05) to WT control mice are indicated by the asterisks.
(TIF)

Figure S3 **Representative micrographs of giemsa-stained cremaster muscle whole mounts of** ***RAGE^−/−^*** **and WT mice with and without PC and aPC treatment.** TNFα-stimulated cremaster muscle whole mounts were obtained after the respective intravital microscopic experiment followed by giemsa-staining. Leukocyte transmigration is illustrated in cremaster muscle of WT (left side) and *RAGE^−/−^* (right side) mice after treatment with saline (A and B), PC (100 U/kg, 3 h; C and D) or aPC (24 µg/kg/h, 3 h; E and F). Reference bar for (A–F) is shown in (A) and represents 50 µm. Arrows indicate neutrophils.
(TIF)

Figure S4 **Comparison of PC and aPC effects on leukocyte adhesion in** ***RAGE^−/−^*** **mice during TNFα-stimulation.** Direct comparison of the relative decrease [%] of leukocyte adhesion in TNFα stimulated cremaster muscle venules after PC (100 U/kg, 3 h) or aPC treatment (24 µg/kg/h, 3 h) in *RAGE^−/−^* mice. All values are presented as mean+SEM from three or more mice per group. Significant differences (*P*<0.05) to PC treated *RAGE^−/−^* mice are indicated by the asterisks.
(TIF)

Table S1 **Hemodynamic Parameters.** Vessel diameter, centerline velocity and wall shear rate of surgically prepared cremaster muscle venules (Trauma) and tumor necrosis factor-α (TNFα)-stimulated cremaster muscle venules of wild-type (WT), and *RAGE^−/−^* mice with protein C (PC) activated protein C (aPC) or saline treatment (control) are presented as mean ± SEM. n.s., not significant.
(DOC)

Table S2 **Coagulation Parameters.** Blood values of INR (international normalized ratio), systemic levels of fibrinogen, activated partial Thromboplastin Time (aPTT) and human Protein C were measured in TNFα-stimulated (500 ng/mouse) WT and *RAGE^−/−^* saline-treated control mice and in WT mice 3 hours after PC administration (100 U/kg) in at least three mice per group, which are presented as mean ± SEM. Significant differences (*P*<0.05) are indicated by an asterisk. n.s., not significant; n.a., not assessed.
(DOC)

Movie S1 **TNFα-induced leukocyte adhesion in WT control mice.** Intravital microscopy was used to visualize leukocyte adhesion in a TNFα-stimulated (500 ng, 3 h) cremaster muscle venule (vessel diameter = 28 µm) of a WT control mouse. TNFα induced significant leukocyte adhesion to the venular wall.
(MPG)

Movie S2 **TNFα-induced leukocyte adhesion in aPC treated WT mice.** Intravital microscopy was used to visualize leukocyte adhesion in a TNFα-stimulated (500 ng, 3 h) cremaster muscle venule (vessel diameter = 27 µm) of a WT mouse after aPC treatment (24 µg/kg/h for 3 h). TNFα-induced leukocyte adhesion was strongly reduced by aPC treatment.
(MPG)

Movie S3 **TNFα-induced leukocyte adhesion in** ***RAGE^−/−^*** **control mice.** Intravital microscopy was used to visualize leukocyte adhesion in a TNFα -stimulated (500 ng, 3 h) cremaster muscle venule (vessel diameter = 24 µm) of a *RAGE^−/−^* control mouse. TNFα- induced leukocyte adhesion in the absence of RAGE is significantly lower compared to the WT mouse.
(MPG)

Movie S4 **TNFα-induced leukocyte adhesion in aPC treated** ***RAGE^−/−^*** **mice.** Intravital microscopy was used to visualize leukocyte adhesion in a TNFα-stimulated (500 ng, 3 h) cremaster muscle venule (vessel diameter = 21 µm) of a *RAGE^−/−^* mouse after aPC treatment (24 µg/kg/h for 3 h). Treatment with aPC led to a further reduction of TNFα-induced leukocyte adhesion compared to the number of adherent leukocytes in the control *RAGE^−/−^* mouse.
(MPG)
