# Supplementary figures and images for: RAGE Controls Activation and Anti-Inflammatory Signalling of Protein C
Source: PLoS One. 2014 Feb 24;9(2):e89422. doi: 10.1371/journal.pone.0089422 (PMC3933550; doi:10.1371/journal.pone.0089422)

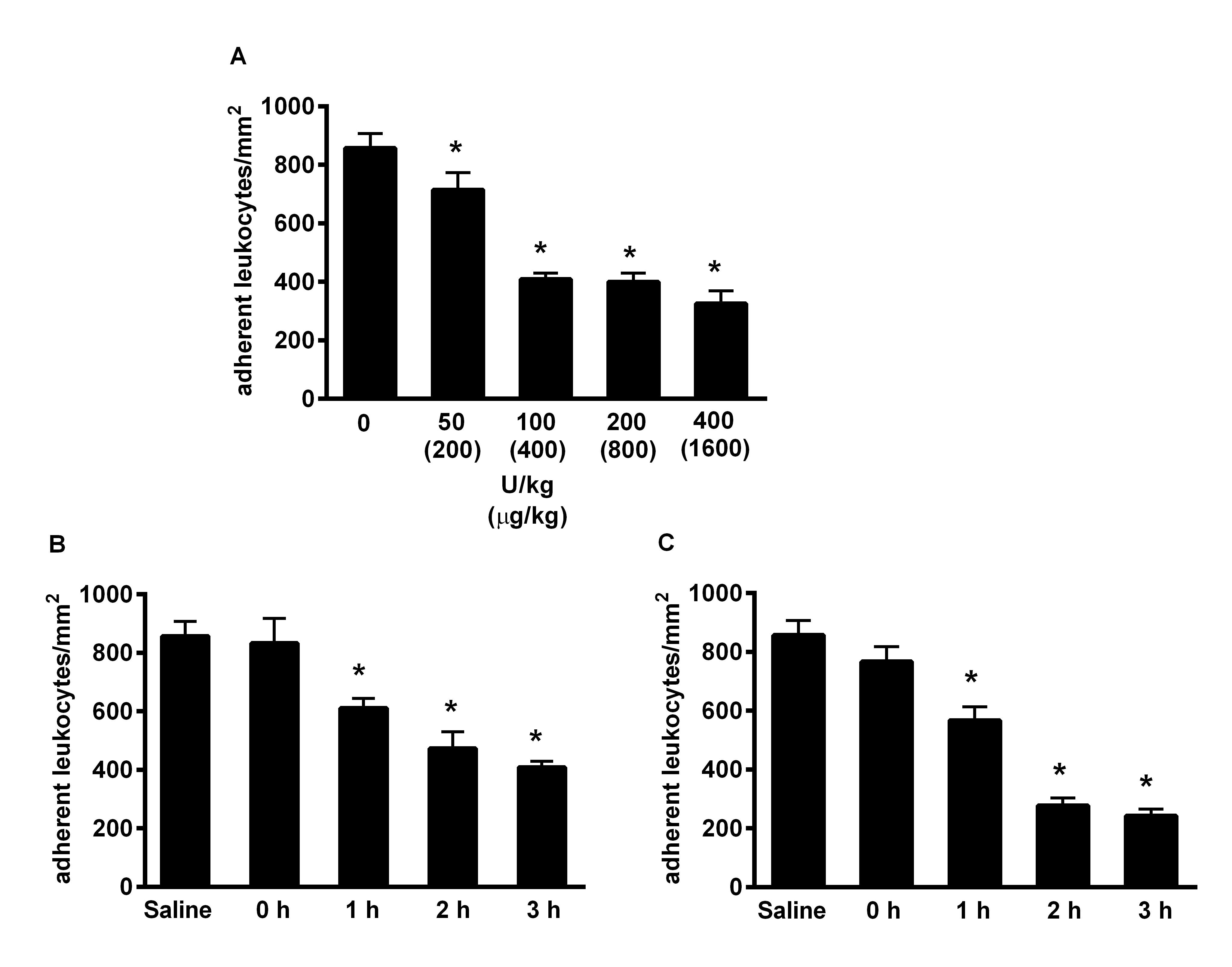

Supplement: Figure S1 — Dose dependent impact of PC (A) on leukocyte adhesion (number of adherent cells per mm2 of surface area) in TNFα (3 h) inflamed cremaster muscle venules of WT mice. Time dependent effect of protein C (PC 100 U/kg; B) and activated protein C (aPC 24 µg/kg/h; C) treatment for leukocyte adhesion in cremaster muscle venules of WT mice were measured after 3 hours TNFα stimulation. All leukocyte adhesion values were obtained by intravital microscopy and are presented as mean+SEM from three or more mice per group. Significant differences (P<0.05) to control mice are indicated by the asterisks. (TIF) [file pone.0089422.s001.tif]

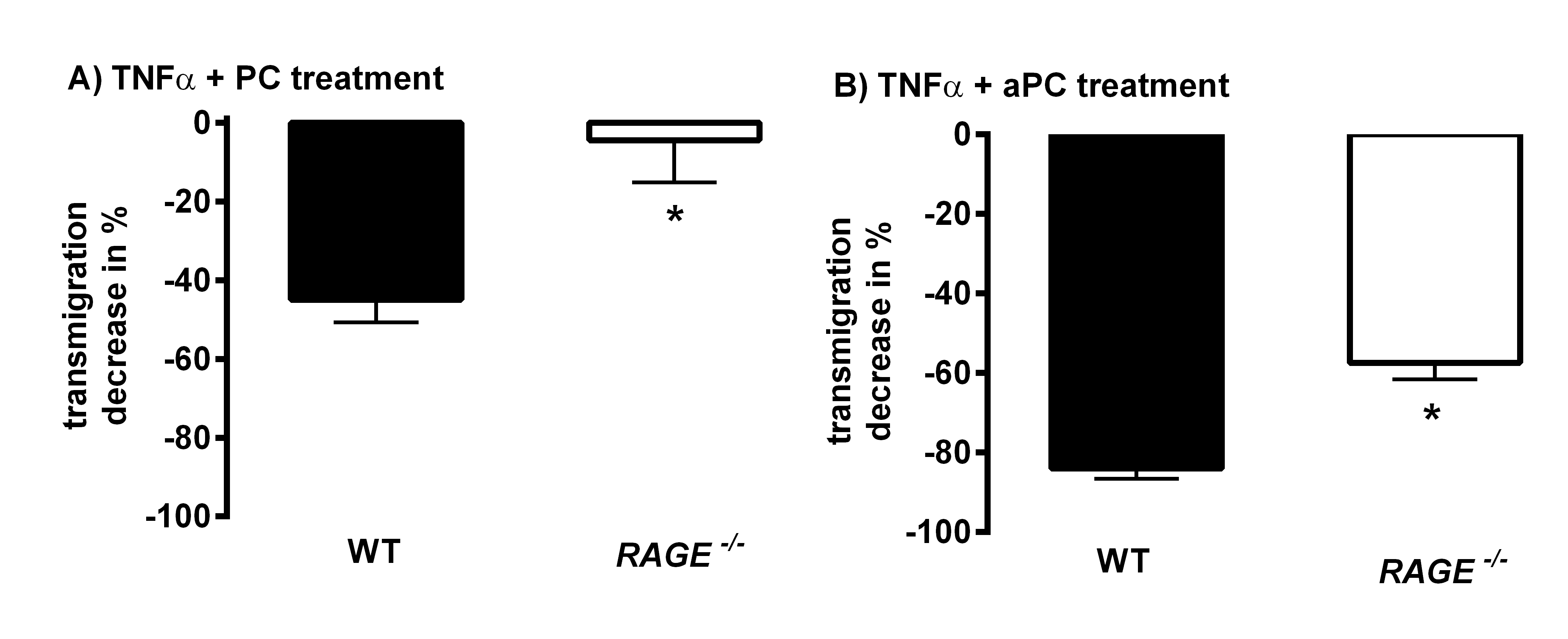

Supplement: Figure S2 — Effect of PC and aPC on leukocyte transmigration in giemsa-stained cremaster muscle whole mounts of RAGE−/− mice compared to WT control mice. Cremaster muscle whole mounts were obtained after the respective intravital microscopic experiment followed by giemsa-staining. Comparison of relative decrease of leukocyte transmigration [%] during TNFα induced inflammation of PC (100 U/kg, 3 h) (A) and aPC (24 µg/kg/h, 3 h) (B) treated WT and RAGE−/− mice. All values are presented as mean+SEM from three or more mice per group. Significant differences (P<0,05) to WT control mice are indicated by the asterisks. (TIF) [file pone.0089422.s002.tif]

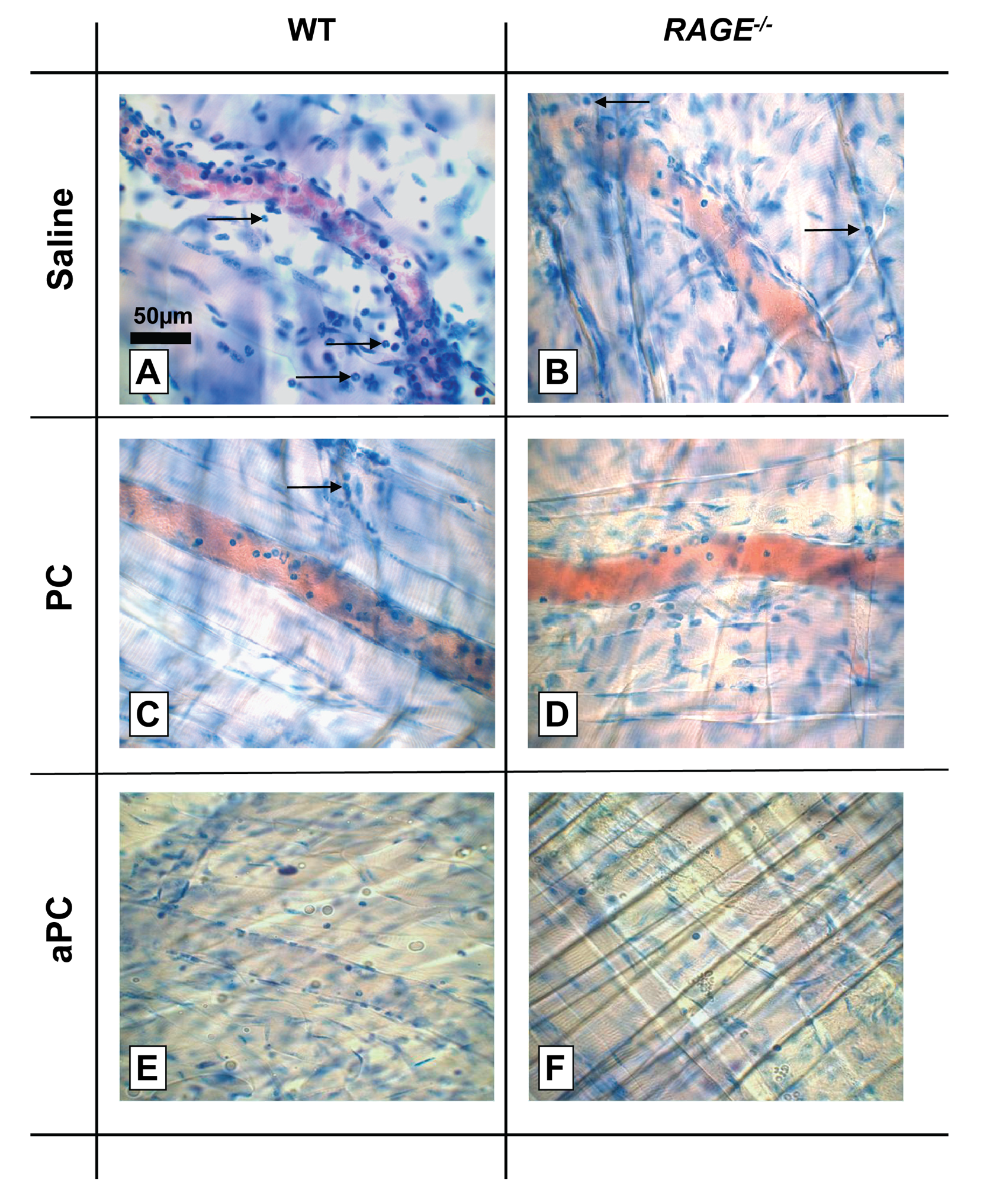

Supplement: Figure S3 — Representative micrographs of giemsa-stained cremaster muscle whole mounts of RAGE−/− and WT mice with and without PC and aPC treatment. TNFα-stimulated cremaster muscle whole mounts were obtained after the respective intravital microscopic experiment followed by giemsa-staining. Leukocyte transmigration is illustrated in cremaster muscle of WT (left side) and RAGE−/− (right side) mice after treatment with saline (A and B), PC (100 U/kg, 3 h; C and D) or aPC (24 µg/kg/h, 3 h; E and F). Reference bar for (A–F) is shown in (A) and represents 50 µm. Arrows indicate neutrophils. (TIF) [file pone.0089422.s003.tif]

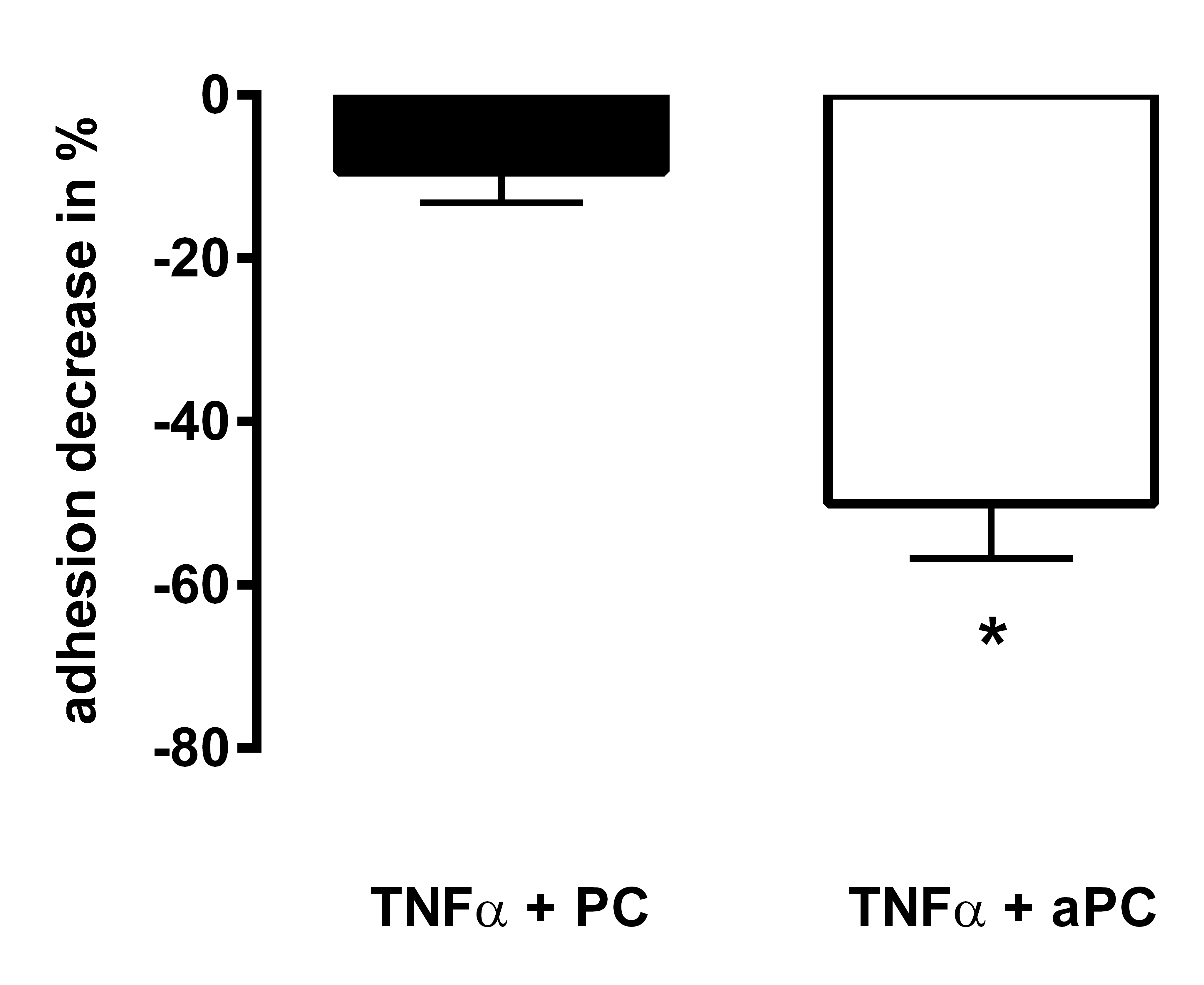

Supplement: Figure S4 — Comparison of PC and aPC effects on leukocyte adhesion in RAGE−/− mice during TNFα-stimulation. Direct comparison of the relative decrease [%] of leukocyte adhesion in TNFα stimulated cremaster muscle venules after PC (100 U/kg, 3 h) or aPC treatment (24 µg/kg/h, 3 h) in RAGE−/− mice. All values are presented as mean+SEM from three or more mice per group. Significant differences (P<0.05) to PC treated RAGE−/− mice are indicated by the asterisks. (TIF) [file pone.0089422.s004.tif]
