# Supplementary material for: RAGE Controls Activation and Anti-Inflammatory Signalling of Protein C
Source: PLoS One. 2014 Feb 24;9(2):e89422. doi: 10.1371/journal.pone.0089422 (PMC3933550; doi:10.1371/journal.pone.0089422)
Supplement: Table S1 — Hemodynamic Parameters. Vessel diameter, centerline velocity and wall shear rate of surgically prepared cremaster muscle venules (Trauma) and tumor necrosis factor-α (TNFα)-stimulated cremaster muscle venules of wild-type (WT), and RAGE−/− mice with protein C (PC) activated protein C (aPC) or saline treatment (control) are presented as mean ± SEM. n.s., not significant. (DOC) [file pone.0089422.s005.doc]

Table S1. Hemodynamic Parameters

|  | *Mice* | *Venules* | *Diameter* | *Centerline Velocity* | *Wall Shear Rate* | | *Systemic Leukocyte Counts* |
| --- | --- | --- | --- | --- | --- | --- | --- |
|  | N | n | (µm) | (µm/s) | (s^-1^) | | (/µl) |
| *Trauma-induced inflammation Genotype/Treatment* | | | | | | | |
| WT control | 11 | 23 | 30 + 1 | 2200 + 100 | 1800 + 100 | 7100 + 300 | |
| WT PC | 11 | 24 | 30 + 1 | 2500 + 100 | 2200 + 100 | 6000 + 400 | |
| WT aPC | 6 | 44 | 28 + 1 | 2200 + 100 | 2100 + 100 | 4100 + 300 | |
| *RAGE^-/-^*control | 12 | 32 | 30 + 1 | 2400 + 100 | 1900 + 100 | 7300 + 800 | |
| *RAGE^-/-^* PC | 9 | 19 | 30 + 1 | 2100 + 200 | 1900 + 100 | 6700 + 1100 | |
| *RAGE^-/-^* aPC | 3 | 24 | 29 + 1 | 2100 + 100 | 1800+ 100 | 4200 + 400 | |
|  |  |  | n.s. | n.s. | n.s. | n.s. | |
| TNF*α-induced inflammation Genotype/Treatment* | | | | | | | |
| WT control | 7 | 55 | 28 + 1 | 2000 + 100 | 1800 + 100 | 2900 + 400 | |
| WT PC | 8 | 62 | 28 + 1 | 2000 + 100 | 1800 + 100 | 3600+ 300 | |
| WT aPC | 3 | 37 | 28 + 1 | 2200 + 100 | 1900 + 100 | 3600 + 500 | |
| *RAGE^-/-^*control | 8 | 38 | 30 + 1 | 2200 + 100 | 2000 + 100 | 3000 + 300 | |
| *RAGE^-/-^* PC | 8 | 47 | 28 + 1 | 2100 + 100 | 2000 + 100 | 3800 + 200 | |
| *RAGE^-/-^* aPC | 5 | 46 | 28 + 1 | 2000+ 100 | 1800 + 100 | 3000 + 300 | |
|  |  |  | n.s. | n.s. | n.s. | n.s. | |
