# Supplementary material for: RAGE Controls Activation and Anti-Inflammatory Signalling of Protein C
Source: PLoS One. 2014 Feb 24;9(2):e89422. doi: 10.1371/journal.pone.0089422 (PMC3933550; doi:10.1371/journal.pone.0089422)
Supplement: Table S2 — Coagulation Parameters. Blood values of INR (international normalized ratio), systemic levels of fibrinogen, activated partial Thromboplastin Time (aPTT) and human Protein C were measured in TNFα-stimulated (500 ng/mouse) WT and RAGE−/− saline-treated control mice and in WT mice 3 hours after PC administration (100 U/kg) in at least three mice per group, which are presented as mean ± SEM. Significant differences (P<0.05) are indicated by an asterisk. n.s., not significant; n.a., not assessed. (DOC) [file pone.0089422.s006.doc]

**Table S2.** **Coagulation Parameters**

| *group* | *Protein C (%)* | *INR* | *Fibrinogen (g/dL)* | *aPTT (s)* |
| --- | --- | --- | --- | --- |
| WT (saline) control | 9,2 ± 1,7 | 0,8 ± 0,1 | 1,2 ± 0,1 | 25,7 ± 2,1 |
| WT PC treatment | 50,6 ± 4,7* | 0,9 ± 0,02 | 0,7 ± 0,2 | 29,9 ± 1,9 |
| *RAGE^-/-^* control | 8,0 ± 0,7 | n.a. | n.a. | n.a. |
|  | *****significant vs. WT & *RAGE^-/-^* control | n.s. | n.s. | n.s. |
